# Case Report: Expansion of the *POLD1*-related polymerase proofreading-associated polyposis spectrum: first report of duodenal adenocarcinomas and characterization of two likely pathogenic variants

**DOI:** 10.3389/fonc.2025.1727289

**Published:** 2025-12-19

**Authors:** Anais Folletet, Morgane Helyon, Maud Privat, Nancy Uhrhammer, Mathilde Gay-Bellile, Mathias Cavaille, Flora Ponelle-Chachuat, Yannick Bidet, Mathis Lepage

**Affiliations:** 1 Département d’Oncogénétique, Centre Jean Perrin, Clermont-Ferrand, France; 2 Digestive Oncology Department, Clermont Ferrand University Hospital, Clermont-Ferrand, France; 3 INSERM, U1240 Imagerie Moléculaire et Stratégies Théranostiques, Université Clermont Auvergne, Clermont-Ferrand, France

**Keywords:** cancer predisposition, DNA replication fidelity, duodenal adenocarcinoma, germline variant, immunotherapy, POLD1, polymerase proofreading-associated polyposis, tumor mutation burden

## Abstract

**Background:**

Polymerase proofreading-associated polyposis (PPAP) is a rare autosomal dominant cancer predisposition syndrome caused by germline pathogenic variants in *POLE* or *POLD1*. While colorectal and endometrial cancers are the most frequent manifestations, the full tumor spectrum of *POLD1*-related PPAP remains incompletely defined.

**Case presentation:**

We describe two families carrying germline *POLD1* variants classified as likely pathogenic. A novel missense variant c.1481T>G p.(Ile494Ser) and a recurrent missense variant c.1204G>A p.(Asp402Asn) were identified within the exonuclease domain. Both variants exhibited features consistent with pathogenicity, including high tumor mutational burden (TMB) and SBS10d mutational signature. Affected carriers developed colorectal and endometrial cancers, but also duodenal adenocarcinomas: this is the first report of this tumor type in germline *POLD1* carriers.

**Conclusions:**

Our report expands both the phenotypic and molecular spectrum of *POLD1*-associated PPAP by documenting the first duodenal adenocarcinomas in germline carriers and describing a novel variant. These findings emphasize the need for systematic upper gastrointestinal surveillance, support the systematic reporting of rare *POLD1* variants to refine genotype–phenotype correlations, and underline the potential therapeutic relevance of identifying carriers in the context of immunotherapy.

## Introduction

The tumor predisposition syndrome associated with *POLD1*, also referred to as PPAP (*Polymerase Proofreading-Associated Polyposis*), is a rare autosomal dominant hereditary cancer predisposition syndrome first described in 2013 (1). It is estimated to account for only 0.1% of all hereditary cancers and approximately 0.3% of colorectal cancer cases associated with polyposis (2). Two genes are known to cause PPAP: *POLD1*, and its analogous gene *POLE*, both implicated in activity of DNA polymerases delta (Polδ) and epsilon (Polϵ) respectively. The tumor spectrum associated with PPAP is similar to that of Lynch syndrome. Classically reported conditions include colorectal polyposis, colorectal cancers, and endometrial cancers. Although the full phenotypic spectrum remains incompletely defined, other neoplasms, such as brain tumors, breast cancer, and duodenal adenomas or carcinomas, may also be associated with this syndrome (1, 3–5).

The *POLD1* gene encodes the catalytic subunit of DNA polymerase delta (Polδ), an enzyme essential for DNA replication. Polδ allows lagging-strand synthesis by elongating Okazaki fragments through its 5’→3’ polymerase activity. Additionally, it has a 3’→5’ exonuclease activity, useful for high-fidelity DNA replication: this is a real-time proofreading mechanism, conferred by its catalytic subunit, POLD1 (6).

The tumor phenotype linked to *POLD1* often includes a high tumor mutational burden (TMB), *POLD1*-specific mutational signatures (SBS10d and SBS10c), and a microsatellite stable (MSS) and/or proficient mismatch repair (pMMR) status. The mutational signatures associated with Polδ deficiency are mostly C>A substitutions occurring in specific nucleotide contexts (5, 7). SBS10d and SBS10c are mainly characterized by TCT > TAT or TCA > TAA substitutions, whereas SBS20 is enriched in CCT > CAT or CCC > CAC substitutions. In rare cases where MSI/dMMR tumors occur with Polδ deficiency, the mutational signature SBS20 may be present (7–9).

Causal variants in *POLD1* are typically pathogenic missense variants within the exonuclease domain that impair exonuclease activity while preserving polymerase function, leading to an increased mutation rate. There is also a distinct pathogenic mechanism in *POLD1* where the polymerase activity is lost, associated with an extremely rare constitutional disorder unrelated to cancer predisposition: Mandibular hypoplasia, Deafness, Progeroid features, and Lipodystrophy (MDPL) syndrome (OMIM: 615381) (10). To date, only nine germline variants have been reported as pathogenic or likely pathogenic in the context of tumor predisposition syndrome (9), reflecting the rarity and specificity of such alterations. In this study, we report a novel germline variant c.1481T>G p.(Ile494Ser) and confirm the pathogenicity of another recurrent variant c.1204G>A p.(Asp402Asn) in *POLD1* (NM_002691.4). The associated tumor spectrum included classically described neoplasms as well as duodenal adenocarcinomas, thereby expanding the phenotypic range of the syndrome.

## Case report

### Clinical report

The index case in Family A is a woman diagnosed with colon adenocarcinoma at age 43, followed by another colon adenocarcinoma at age 69. She was also diagnosed with endometrial adenocarcinoma at age 54, and with bifocal hormone receptor-positive invasive ductal carcinoma of the breast at age 60. Colonic polyps were detected at each colonoscopy during her follow-up. At least 40 polyps were resected since age of 41, with predominance of either adenomas with low grade dysplasia, or serrated polyps. All colonic segments were affected. The MMR status of the various tumors could not be assessed, as the patient had been treated at a private clinic where tumor blocks are only retained for 10 years. However, two polyps were MSS.

Her maternal half-brother died from complications of colon adenocarcinoma diagnosed at the age of 32. The son of this half-brother, also deceased, was diagnosed at age 52 with synchronous double malignancies. These included an infiltrating duodenal adenocarcinoma extending into the pancreas (deficient-MMR: isolated loss of PMS2) and a colonic adenocarcinoma, in the context of polyposis. He himself had a daughter who developed 4 duodenal and colonic polyps at the age of 23: two duodenal tubular adenomas with low grade dysplasia, a 1 cm tubulovillous adenoma with high-grade dysplasia (pMMR), and a tubular adenoma near anal margin. Another maternal half-brother died after being diagnosed with colon adenocarcinoma at 60, followed by bladder cancer, a malignant tumor of the ampulla of Vater, and pulmonary squamous cell carcinoma (in the context of past smoking) around the age of 80. Finally, another maternal half-sister, also deceased, developed colonic polyps, endometrial adenocarcinoma at 66, rectal adenocarcinoma at 67, and a second rectal adenocarcinoma at 83. The daughter of this half-sister was diagnosed with colon adenocarcinoma (pMMR) at 53.

Their mother developed rectal cancer at the age of 51 and died at 66. It was not possible to determine if she had polyposis. Their maternal aunt and uncle both apparently had colon cancer as well.

The index case of Family B is a man diagnosed with duodenal adenocarcinoma (pMMR) at the age of 40. The esophagogastroduodenal exploration and colonoscopy did not reveal any other lesions. When the likely pathogenic *POLD1* variant was identified, his oncologist planned to discuss the potential benefits of immunotherapy at a multidisciplinary team meeting upon completion of the staging of his duodenal cancer.

His mother died at 66 from metastatic lung cancer. No additional neoplastic history has been reported in this family to date. None of his relatives have undergone genetic testing currently.

**Figure 1:**
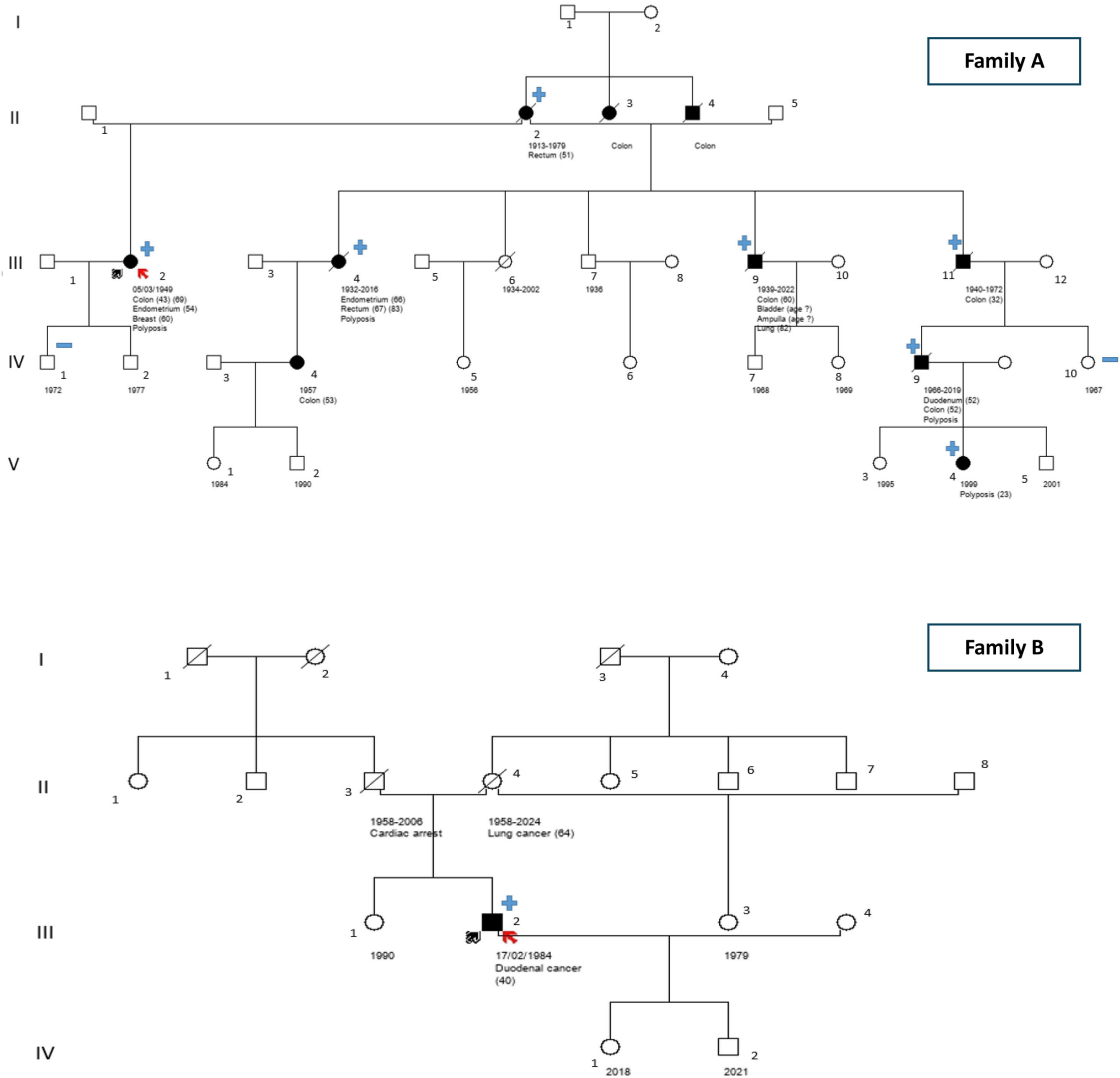
Pedigrees of the two POLD1 families identified in our study.

Pedigrees for both families are shown in **Figure 1**.

### Variant classification

An NGS analysis of the gastrointestinal panel recommended by the French Genetics and Cancer Group was performed on two individuals from family A (III.2 and V.4) and on the index case from family B (III.2) (11). Two *POLD1* germline variants were identified, both of which were classified as likely pathogenic (ACMG class 4) according to current recommendations (12, 13): NM_002691.4: *POLD1:*c.1481T>G p.(Ile494Ser) – Family A and NM_002691.4: *POLD1*:c.1204G>A p.(Asp402Asn) – Family B. No other class 3, 4 and 5 variants were identified in the analyzed genes (*APC, BMPR1A, CDH1, EPCAM, MLH1, MSH2, MSH6, MUTYH, PMS2, POLD1, POLE, PTEN, SMAD4* and *STK11)*.

The *POLD1*:c.1481T>G p.(Ile494Ser) variant is absent from the general population database gnomAD v4 and from literature (PM2_Supporting). The variant is located in the *POLD1* exonuclease domain, just outside the ExoV motif (positions 485–493), affecting the immediately adjacent amino acid; thus, there is proximity to a functionally critical motif (PM1_Supporting). *In silico* prediction tools indicate a deleterious effect, with a REVEL score of 0.654 (above the 0.644 threshold) (PP3). Co-segregation analysis demonstrated at least six informative meioses, exceeding the threshold of five (PP1_Moderate). Tumor analysis of a polyp resected in 2019 (Family A, patient III.2) revealed a TMB-high phenotype (27.1 Mut/Mb), an SBS10d mutational signature, and pMMR status (PP4_Moderate). Consequently, the c.1481T>G variant is classified as likely pathogenic (PM2_Sup + PM1_Sup/PP3 + PP1_Mod + PP4_Mod).

The *POLD1*:c.1204G>A p.(Asp402Asn) variant is extremely rare in the gnomAD v4 database, with only one allele reported (PM2_Supporting). The variant lies within the exonuclease domain of *POLD1*, in the ExoII motif (positions 393–406) (PM1_Supporting). Furthermore, according to data from literature (14), tumor analysis in a patient with endometrial cancer carrying this germline variant showed a TMB-high phenotype (141 Mut/Mb), an SBS10d mutational signature, a pMMR status and loss of heterozygosity (LOH) at *POLD1* (PP4_Strong). The c.1204G>A variant is also classified as likely pathogenic (PM2_Sup + PM1_Sup + PP4_Strong). Moreover, one functional study using site-directed mutagenesis in *S. cerevisiae* has demonstrated a deleterious effect (15) (PS3_Sup). This variant has been reported in ClinVar once as likely pathogenic and five times as a variant of uncertain significance.

### Literature review

**Table 1** summarizes *POLD1* germline variants located within the exonuclease domain and reported in the literature as pathogenic or likely pathogenic. The exonuclease domain can be divided into several exonuclease (Exo) motifs:

**Table 1:** Currently reported POLD1 germline pathogenic/likely pathogenic variants within the exonuclease domain.

Nucleotide change	Amino acid change	Exo Motif	Tumor spectrum in heterozygotes	Somatic analysis if available	Number of affected families	References
c.946G>C	p.(Asp316His)	ExoI	CRC, BC, Mesothelioma, Angiomyolipoma	pMMR	1	(4)
c.946G>A	p.(Asp316Asn)	ExoI	CRC, EC, BC, UroC, Nephrogenic adenoma → Co-inheritance with PMS2 (CMMRD-like)	dMMR TMB-high (278 and 379 Mut/Mb) SBS20	1	(16)
c.947A>G	p.(Asp316Gly)	ExoI	CRC, EC, BC	pMMR	1	(4)
c.952G>A	p.(Glu318Lys)	ExoI	EC, BrT	n/a	1	(17)
			CRC	n/a	1	(18)
c.1204G>A	p.(Asp402Asn)	ExoII	CRC	n/a	1	(18)
			EC	pMMR TMB-high (141 Mut/Mb) SBS10d	1	(14)
			DuoC	pMMR	1	This study
c.1225C>T	p.(Arg409Trp)	n/a	CRC	pMMR	1	(4)
c.1421T>C	p.(Leu474Pro)	ExoIV	CRC, EC, GIST	pMMR	1	(3)
			CRC	pMMR	1	(4)
			CRC, EC, BC, GIST	pMMR and dMMR	4	(19)
			CRC	n/a	1	(20)
c.1433G>A	p.(Ser478Asn)	ExoIV	CRC, EC, BrT	pMMR	2	(1)
			CRC, EC, BrT	pMMR	1	(21)
			CRC	n/a	1	(22)
			CRC	n/a	1	(23)
			CRC, EC	n/a	1	(24)
			CRC, EC, BrT	pMMR	1	(25)
c.1458G>T	p.(Lys486Asn)	ExoV	CRC, EC, OvC, PrC	pMMR	2	(5)
c.1481T>G	p.(Ile494Ser)	n/a	CRC, EC, DuoC, AmpC, UroC, BC, LC	pMMR TMB-high (27.1 Mut/Mb) SBS10d	1	This study

CRC, Colorectal cancer, EC, Endometrial cancer, BrT, Brain tumors, GIST, Gastro-intestinal stromal tumor, BC, Breast cancer, OvC, Ovarian cancer, PrC, Prostate cancer, UroC, Urothelial cancer, DuoC, Duodenal cancer, AmpC, Malignant tumor of the am, lla of Vater.

ExoI (aa 312-326), ExoII (393–406), ExoIV (aa 470-478), ExoV (485–493) and ExoIII (506–519).

From the literature, we identified 9 germline variants reported across 15 studies, focusing on those classified as pathogenic or likely pathogenic within the exonuclease domain. Eight of these variants were located within Exo motifs, with all motifs represented except ExoIII. Positioning within an Exo motif is considered an additional criterion supporting pathogenicity in current classification systems. Four variants have been reported in at least two independent studies, and all of them are situated within Exo motifs.

While most pathogenic or likely pathogenic *POLD1* variants cluster within the exonuclease domain, often in or near Exo motifs, at least one germline alteration has been described outside this region, in the polymerase domain c.1816C>A p.(Leu606Met), further broadening the mutational spectrum of the gene. This variant, reported as likely pathogenic (26), was identified in a 22-year-old woman diagnosed with gastrointestinal polyposis, breast fibroadenoma, multiple primary colorectal cancers, and glioblastoma (grade IV) within 4 years. Both rectal cancer and glioblastoma affected tissues exhibited a TMB-high phenotype, with 16.9 Mut/Mb and 347.1 Mut/Mb respectively, and were pMMR. The patient had no family history of cancer, and testing of both parents confirmed a *de novo* germline event (validated by genetic fingerprint analysis). No signs of mandibular hypoplasia, deafness, progeroid features, and lipodystrophy syndrome were reported in the article.

This same variant c.1816C>A p.(Leu606Met) was independently reported in ClinVar by Hereditary Cancer Clinic from Medical College of Georgia in 2022. In this second family, the mother is a likely mosaic carrier (variant allele frequency estimated at 14%). She reportedly developed multiple gastrointestinal adenocarcinomas, while two heterozygous daughters presented gastrointestinal polyps. Analysis of some adenomas revealed a high TMB.

## Discussion

In this study, we identified two likely pathogenic *POLD1* variants, namely c.1481T>G p.(Ile494Ser), which has not been previously reported, and c.1204G>A p.(Asp402Asn), a variant with conflicting classifications in the literature (uncertain significance/likely pathogenic). Both variants were classified as likely pathogenic according to gene-specific variant recommendation for non-disruptive variants located in the exonuclease domain of *POLD1* (12), and this interpretation was independently validated by the French expert group on genetics and cancer (Groupe Génétique et Cancer, GGC).

Penetrance in PPAP may differ between *POLD1* and *POLE*. For colorectal cancer, the estimated risk by age 70 is around 90% in *POLE* heterozygotes versus 50% in *POLD1* carriers. Conversely, the risk of endometrial cancer by age 70 seems higher in *POLD1* (75%) than in *POLE* (25%) (20). The newly described *POLD1*:c.1481T>G p.(Ile494Ser) variant appears highly penetrant, as illustrated by family A: all carriers developed colorectal cancer between ages 32 and 66, with possible prior polyposis in some individuals. Endometrial cancer was also diagnosed in two women at 54 and 66 years. Assessing the penetrance of c.1204G>A p.(Asp402Asn) remains challenging, owing to the limited number of carriers identified so far.

The tumor spectrum in PPAP also varies depending on the causal gene, *POLD1* or *POLE*. Colorectal and endometrial cancers are the most frequent manifestations, while other tumors, such as ovarian, breast, brain, and upper gastrointestinal cancers, have occasionally been described but remain less well defined. Until now, duodenal cancers had been described exclusively in *POLE* heterozygotes (9, 27, 28), a discrepancy that may partly reflect the smaller number of reported *POLD1* families compared with *POLE*. With our observations, the associated tumor spectrum is consistent with previous descriptions of PPAP, predominantly involving colorectal and endometrial cancers. However, additional tumor types were reported, most notably duodenal adenocarcinomas in both *POLD1*-positive families. While duodenal adenomas have been associated with germline *POLD1* variants in the past (20), this represents, to our knowledge, the first report of duodenal adenocarcinomas linked to constitutional *POLD1* alterations.

Surveillance of PPAP has been discussed in the literature: some authors recommend initiating colonoscopy as early as age 14 (20, 29), while the National Comprehensive Cancer Network (NCCN, version 1.2025) advises starting between ages 25 and 30, despite reporting a case of colorectal cancer diagnosed at age 14. Upper gastrointestinal (GI) endoscopy is recommended beginning at age 25. Our findings highlight the importance of systematic upper GI surveillance in patients with *POLD1* mutations.

Gynecologic monitoring should also be considered in later adulthood. Palles et al. suggest risk-reducing hysterectomy and bilateral salpingo-oophorectomy; however, current data are insufficient to accurately estimate the risk of ovarian cancer (20). To date, no dedicated screening exists for other PPAP-associated cancers, although vigilance is warranted, particularly regarding brain tumors.

The small number of published *POLD1* families continues to limit risk estimates and hinders precise genotype–phenotype correlations. Collecting additional cases will therefore be critical to refine cancer risks and penetrance estimates, and guide clinical management. Importantly, identifying *POLD1* carriers also has direct therapeutic implications: the proofreading defect underlying PPAP may confer enhanced responsiveness to immunotherapy, representing a major opportunity in cancer treatment (30).

Finally, it is noteworthy that a recently reported likely pathogenic *POLD1* variant c.1816C>A p.(Leu606Met) lies within the polymerase domain. This finding challenges the previous assumption that pathogenic variants for hereditary cancer are confined to the exonuclease domain. Therefore, we would recommend that, in suspected cases of PPAP, the entire *POLD1* gene should be analyzed. Given the rarity of *POLD1* alterations, classification remains difficult, as few consensus criteria are currently established. Ongoing collaborative efforts will be crucial to refine classification frameworks and provide accurate and clinically relevant interpretation of these variants as our understanding of *POLD1*-associated cancer predisposition evolves.

## Conclusion

This study provides the first report of duodenal adenocarcinomas in *POLD1* carriers, expanding the clinical spectrum of *POLD1*-related PPAP. Together with the description of a novel germline variant and confirmation of a recurrent one, these findings reinforce the need for systematic upper gastrointestinal endoscopic follow-up in PPAP, highlight the importance of comprehensive variant reporting to refine genotype-phenotype correlations and risk estimates, thereby informing tailored surveillance guidelines, and point to the therapeutic relevance of identifying carriers in the era of immunotherapy.

## Methods

### Germline analysis

Samples of peripheral blood were used for genomic DNA extraction (Qiagen). Libraries were prepared using KAPA HyperPlus Kits and captured using KAPA HyperCap Target Enrichment (Roche). The quality of libraries and captures was controlled with a Tapestation 4150 (Agilent). Germline sequencing was performed with a NextSeq 550 Instrument (Illumina) using a NextSeq High-Output v2.5 kit (300 cycles).

De‐multiplexing was performed using bcl2fastq2 Conversion Software (Illumina). Alignment was performed on University of California Santa Cruz human genome reference build 19 using the Burrows‐Wheeler Aligner. Genome Analysis Toolkit (GATK) and PICARD tools were used for base quality score recalibration (BaseRecalibrator) and realignment (RealignerTargetCreator, IndelRealigner. Variant calling was performed using GATK HaplotypeCaller and annotated using Ensembl Variant Effect Predictor. CNV analysis was performed using ExomeDef. Variants were filtered for quality score ≥ 30, and present in ≥ 20% of reads. All class 4 variants were confirmed on a second independent sample by the Sanger technique using a 3500xl instrument and BigDye terminator kit 3.1 (Applied Biosystems). Sanger was used to test the relatives.

The entire coding sequence and intron/exon junctions (positions up to -50 and + 20) of the 14 genes recommended by the French Genetics and Cancer Group for the panel analysis in hereditary predispositions to tumors of the digestive tract had a minimum depth of coverage of 30X (*APC, BMPR1A, CDH1, EPCAM, MLH1, MSH2, MSH6, MUTYH, PMS2, POLD1, POLE, PTEN, SMAD4* and *STK11)*, except for exons 11 to 15 of PMS2, due to high identity with paralogous genes (11). Raw data are available in the supplementary documents.

### Somatic analysis

The polyp analysis was outsourced to the French laboratory of reference at the Curie Institute. Targeted DNA sequencing was performed from FFPE samples using a custom NGS panel including more than 500 genes (marketed by Agilent under the name of SureSelect CD Curie CGP). The samples were processed as described in previously published protocol (31).

## Data Availability

The raw data supporting the conclusion of this article are included in the supplementary material. Further inquiries can be directed to the corresponding author.
