# Supplementary figures and images for: Case Report: Expansion of the POLD1-related polymerase proofreading-associated polyposis spectrum: first report of duodenal adenocarcinomas and characterization of two likely pathogenic variants
Source: Front Oncol. 2025 Dec 19;15:1727289. doi: 10.3389/fonc.2025.1727289 (PMC12757262; doi:10.3389/fonc.2025.1727289)

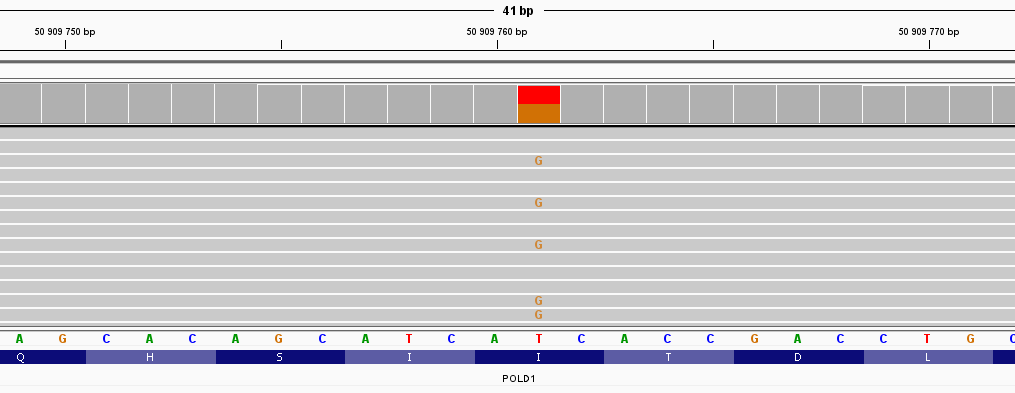
NM_002691.4: POLD1:c.1481T>G p.(Ile494Ser)


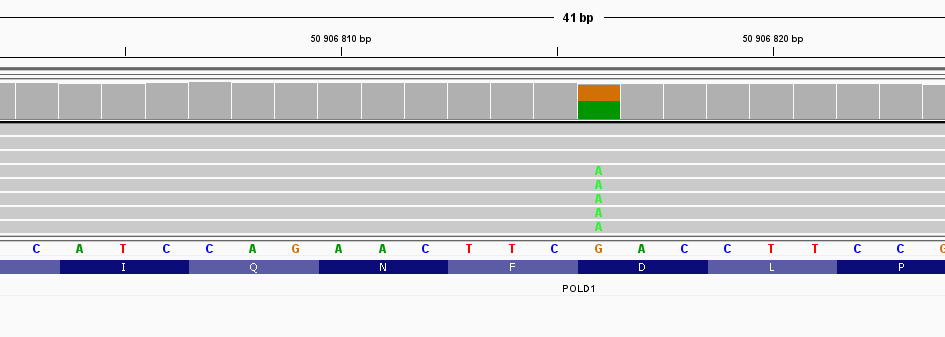
NM_002691.4: POLD1:c.1204G>A p.(Asp402Asn)

Supplement: Supplementary file 1 [file DataSheet1.docx]

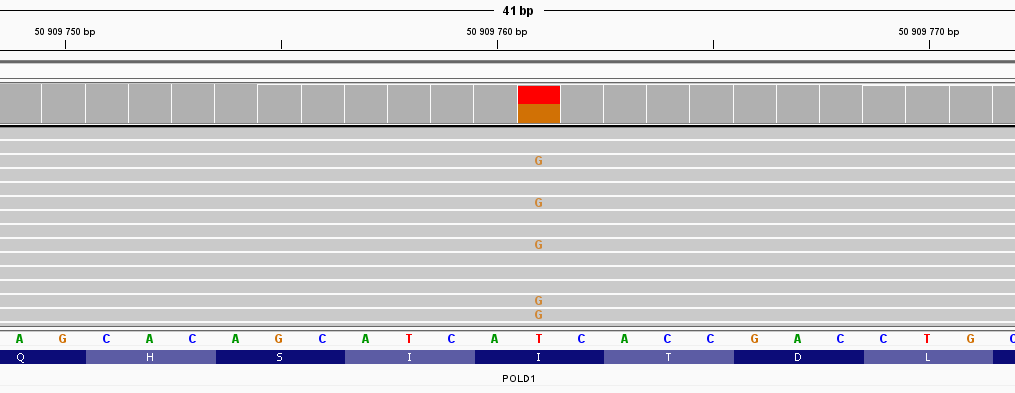

Supplement: Supplementary file 2 [file Image1.png]

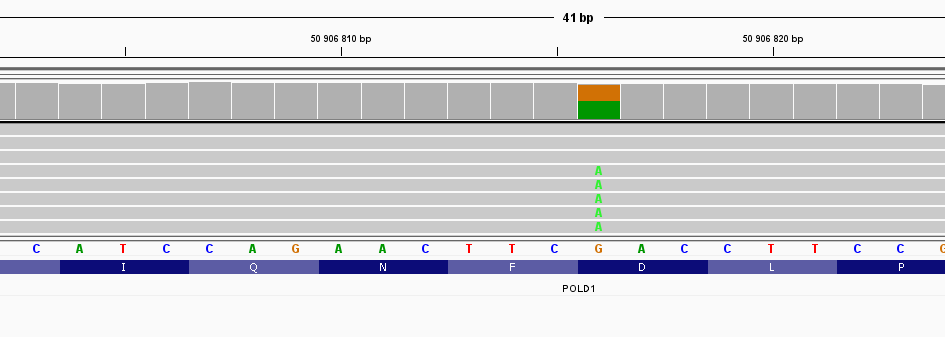

Supplement: Supplementary file 3 [file Image2.png]
